# Cardiovascular Toxicity of Carfilzomib: The Real-World Evidence Based on the Adverse Event Reporting System Database of the FDA, the United States

**DOI:** 10.3389/fcvm.2021.735466

**Published:** 2021-09-27

**Authors:** Yinghong Zhai, Xiaofei Ye, Fangyuan Hu, Jinfang Xu, Xiaojing Guo, Yang Cao, Zhen Lin, Xiang Zhou, Zhijian Guo, Jia He

**Affiliations:** ^1^School of Medicine, Tongji University, Shanghai, China; ^2^Department of Health Statistics, Second Military Medical University, Shanghai, China; ^3^Clinical Epidemiology and Biostatistics, School of Medical Sciences, Örebro University, Örebro, Sweden

**Keywords:** cardiovascular toxicity, carfilzomib, FAERS, disproportionality analysis, real-world evidence

## Abstract

**Background:** Carfilzomib, an effective proteasome inhibitor agent for the therapy of relapsed and refractory multiple myeloma, has been related to a significant number of cardiovascular events. However, patterns of cardiovascular complications associated with this agent remain poorly characterized in real-world settings.

**Objective:** To gain further insight into the frequency, spectrum, clinical features, timing, and outcomes of carfilzomib-related cardiovascular toxicities.

**Methods:** This disproportionality (case/non-case) study was conducted leveraging records from FAERS database from 2014 to 2019. Cardiovascular events were defined and broadly categorized eight entities using narrow version of the Standardized MedDRA Queries (SMQs). Reporting odds ratios (ROR) and information component (IC) were calculated to measure disproportionality. Additionally, statistical shrinkage was applied to reduce false-positive signals.

**Results:** The final number of records involved was 28,479,963, with 3,370 records submitted for carfilzomib related cardiovascular events. Significant disproportionality association between carfilzomib administration and cardiovascular events was captured (IC_025_/ROR_025_ = 0.85/1.95) when exploring in the entire database. Upon further analysis, all eight broad categories of cardiovascular toxicities were disproportionately associated with carfilzomib with varying frequencies, time-to-onset, and severities. Cardiomyopathy-related complications (*N* = 1,301, 38.61%), embolic and thrombotic events (*N* = 821, 24.36%), and cardiac failure (*N* = 765, 22.70%) largely comprised the reported problems. Notably, the strongest signal was detected for cardiac failure (IC_025_/ROR_025_ = 1.33/2.59), followed by pulmonary hypertension (IC_025_/ROR_025_ = 1.19/2.34). Median onset time of cardiovascular events was 41days (Q1-Q3: 9-114 days), with the shortest median time being 16 days (Q1–Q3: 4–85 days) for ischemic heart disease, with the longest time being 68 days (Q1–Q3: 21–139 days) for embolic and thrombotic events. Torsade de pointes/QT prolongation was identified as a new complication (IC_025_/ROR_025_ = 0.33/1.29) and was particularly noteworthy for highest death proportion (44.11%).

**Conclusions:** Treatment with carfilzomib can lead to severe and versatile cardiovascular events. Early and intensive monitoring is important, particularly in the first 3 months after carfilzomib initiation. Maximizing the benefit while reducing potential cardiovascular harms of carfilzomib should become a priority.

## Introduction

Multiple myeloma (MM), despite rare, is the second most common hematologic malignancy worldwide, with estimated 159 985 new cases and 106 105 deaths in 2018 (1). MM remains uncurable but the survival of MM patients has substantially improved with the clinical development of novel therapeutics (2). Proteasome inhibitor (PI) has been widely used in treating MM and become the backbone therapy for MM both in the first- and second-line settings (3). Carfilzomib, authorized in 2012, is a second-generation PI that irreversibly inhibits the chymotryptic site of the proteasome (4). Importantly, carfilzomib is the first and only MM treatment that prolongs overall survival in relapsed settings over the current standard therapy (5).

Carfilzomib has a more favorable safety profile than other PI agents and is used with increasing frequency in clinical practice to treat MM sufferers. However, of the PIs used, carfilzomib is the most strongly associated with cardiotoxicity (6). Although most cardiovascular complications are reversible, in some cases they may lead to life-threatening and even fatal complications (7, 8). Whereas, evidence based on real-world data is sparse and the cardiovascular safety profile of carfilzomib remains incompletely characterized. Therefore, we conducted a pharmacovigilance study based on the FDA Adverse Events Reporting System (FAERS) database to explore the real-world pattern of cardiovascular diseases occurring secondary to carfilzomib in detail. Our study may update the currently described cardiovascular complications of carfilzomib, as well as bring important clues for further study.

## Methods

### Study Design and Data Sources

This observational study with focus of disproportionality analysis was based on a subset of the FAERS database, covering the period from January 2015 to December 2019. FAERS is a spontaneous database maintained by the United States (U.S.) FDA and gathers information on adverse event (AE) reports that originate from different sources, including healthcare professionals, patients, and drug manufacturers (9). The large quantity of the data collected at a national level from the general population allows FAERS to be a useful tool in detecting post-marketing signals of risk for AE. The FDA updates FAERS files every quarter online and all data used in the current study can be accessed at https://fis.fda.gov/extensions/FPD-QDE-FAERS/FPD-QDE-FAERS.html.

### Data Cleaning

The following data cleaning procedure was conducted before the disproportionality analysis. First, we securitized the extracted records according to similarities in demographic characteristics, and drug- and AE-related information. Records with the same sex and age and similar drug name, AE, starting date, reporting year, country of reporter, event date, end date, and outcome were considered duplicated and removed accordingly. Second, records with starting date of drug later than the occurring date of AE were regarded aberrant and excluded from the study (10). No imputations were conducted for missing data, as variables in the FAERS database was missing at a large proportion. Time-to-onset was defined as the period between the start date of carfilzomib and the onset date of cardiovascular events. Severe outcomes were defined as causing any of the following conditions: death, life-threatening, hospitalization, disability, congenital anomaly, requiring intervention to prevent permanent damage, and any other important medical events (11).

Both brand and generic names were used to identify records related to the target drug, carfilzomib. In FAERS, AEs are coded using the preferred term (PT) according to the Medical Dictionary for Regulatory Activities (MedDRA). A specific PT can be assigned to several high-level terms (HLTs), high-level group terms (HLGTs), and system organ classes (SOCs) (12). Additionally, different PTs can be grouped into meaningful broader categories through Standardized MedDRA Queries (SMQs) to define a medical condition or area of interest (13). The hierarchical and multiaxial structure of MedDRA allows flexibility in AE retrieval. In this study, we captured and categorized cardiovascular entities using eight narrow categories of SMQs (cardiac failure, cardiomyopathy, hypertension, pulmonary hypertension, ischemic heart disease, torsade de pointes/QT prolongation, cardiac arrhythmias, embolic and thrombotic events) (Table 1) according to a published study (10). More details of the SMQs used in our study to identify cardiovascular events can be accessed in supplementary materials (Tables S1–S8).

**Table 1:** Cardiovascular events grouping as 20 broad entities according to MedDRA 22.0.

**SMQ name**	**SMQ code**	**Algorithm**
Cardiac arrhythmias	20000049	Narrow
Cardiac failure	20000004	Narrow
Cardiomyopathy	20000150	Narrow
Embolic and thrombotic events	20000081	Narrow
Hypertension	20000147	Narrow
Ischemic heart disease	20000043	Narrow
Pulmonary hypertension	20000130	Narrow
Torsade de pointes/QT prolongation	20000001	Narrow

### Statistical Analysis

Currently, the most common signal detection method is disproportionality (case/non-case) analysis, which was originally developed to evaluate associations between the use of specific drugs and potential AEs (14). Disproportionality emerges when the reporting frequency of a specific AE is highlighted when compared with background data. In this study, the degree of disproportionality was measured through reporting odds ratio (ROR) and information components (IC), which were both calculated through analysis of observed-to-expected reporting frequencies (12, 15), and the expected frequencies was calculated as:


$$\begin{array}{l} n_{Expected}=\left(n_{Drug}\;*\;n_{Event}\right)/n_{Total} \end{array}$$

where *n*_*Expected*_ denoted the number of records expected for the target drug-AE combination, *n*_*Drug*_ and *n*_*Event*_ denoted the total number of records submitted for the selected drug and AE, respectively, and *n*_*total*_ represented the total number of records involved in the entire database. In addition, we applied the statistical shrinkage transformation to address the shortcoming that the two algorithms were sensitive to random fluctuations for rare events (16). The statistical shrinkage transformation is originally proposed by the World Health Organization Uppsala Monitoring Center to reduce false-negative adverse signals. It has been proved that the statistical shrinkage can protect against spurious associations and obtain robust results. In recent years, this statistical technique has been applied more and more in signal detection of adverse drug reactions (11, 17–19). In the present study, the shrunk IC and ROR were calculated as:


$$\begin{array}{l} IC=\log_{2}\left[\left(n_{Observrd}+0.5\right)/\left(n_{Expected}+\;0.5\right)\right]\\ ROR=\left(n_{Observrd}+0.5\right)/\left(n_{Expected}+0.5\right) \end{array}$$

where *n*_*Observrd*_ denoted the number of records observed for the target drug-AE combination.

A signal was determined when the lower limit of 95% confidence interval (CI) of ROR (ROR_025_) exceeded 1 and the corresponding IC (IC_025_) crossed over 0, with at least three records. The characteristics of the associated records were described in terms of means or medians for continuous variables, and frequency and percentages for categorical ones. All data cleaning and statistical analyses were conducted using SAS 9.4 (SAS Institute, Cary, NC).

## Results

### Selection Results

**Figure 1:**
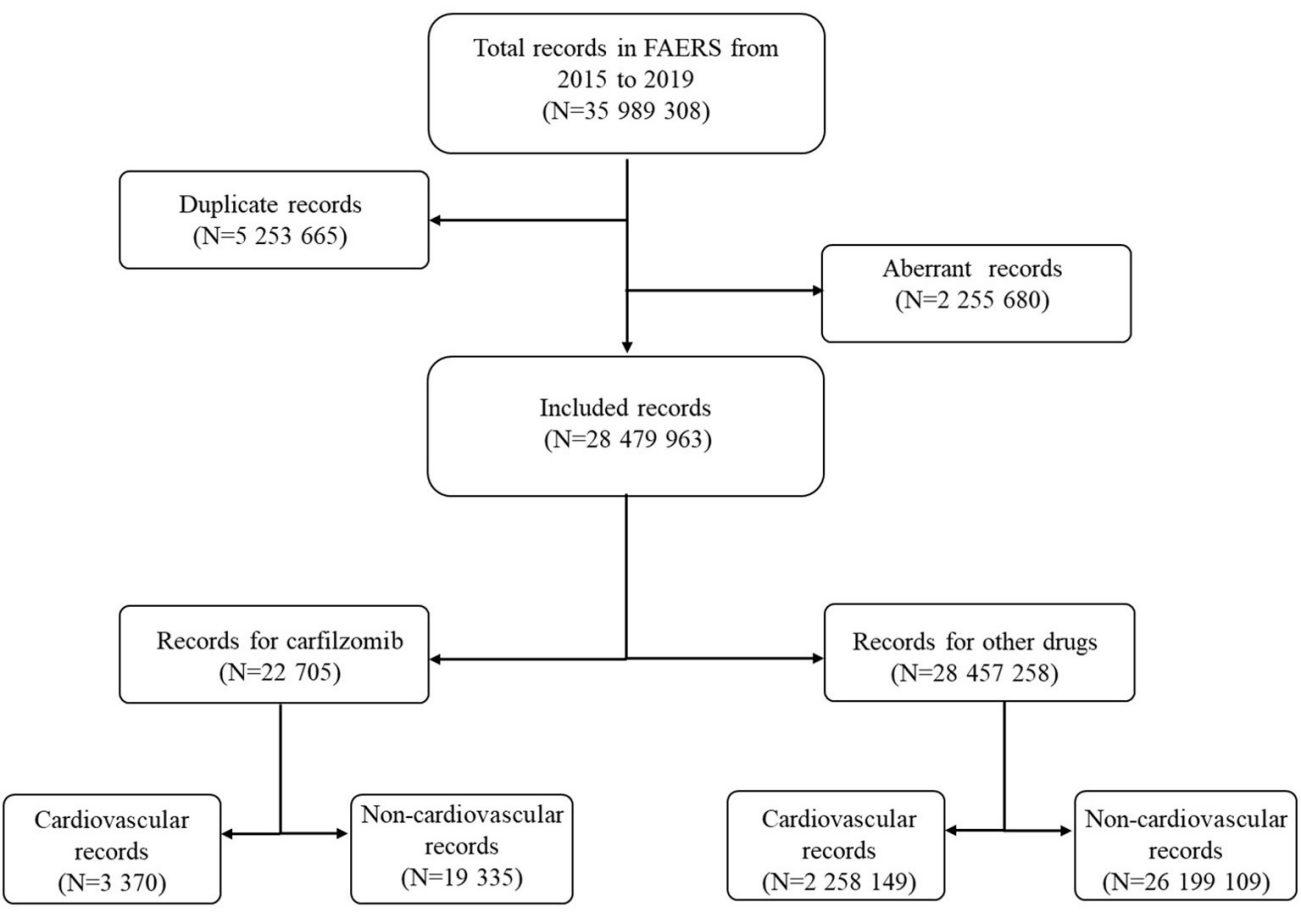
Flow chart of the record selection process.

Over the study period, a total of 35,989,308 records were extracted from the FAERS database (Figure 1). After exclusion of duplicate and aberrant records, the final number of records for analysis was 28,479,963. Twenty two thousand seven hundred and five records were submitted for carfilzomib; of these, a total of 3,370 records were reported for cardiovascular complications.

### Clinical Characteristics

#### Baseline Characteristics

The carfilzomib-related cardiovascular records were mainly from the U.S. (1,586, 47.06%), Germany (409, 12.14%), and Japan (306, 9.08%) (Table 2). After exclusion of 1,029 records with unspecified age, the mean onset age across all records was 66.68 years. The maximum number of records was cardiomyopathy-related complications (1,301, 38.61%), followed by embolic and thrombotic events (821, 24.36%), and cardiac failure (765, 22.70%). Excluding 467 records without missing sex, male accounted for a larger proportion than female, no matter for total or class-specific cardiovascular AEs (Table 2).

**Table 2:** Characteristics of cardiovascular reocrdes associated with carfilzomib in FAERS from 2015–2019.

**Characteristics**	**Total**	**Cardiac Arrhythmias**	**Cardiac Failure**	**Cardiomyopathy**	**Embolic Thrombotic Events**	**Hypertension**	**Ischemic Heart Disease**	**Pulmonary Hypertension**	**Torsade de pointes/QT prolongation**
**Sex**									
Male	1,729 (59.56)	371 (68.20)	426 (63.77)	661 (60.81)	388 (52.01)	115 (56.65)	156 (70.59)	313 (59.73)	183 (65.83)
Female	1,174 (40.44)	173 (31.80)	242 (36.23)	426 (39.19)	358 (47.99)	88 (43.35)	65 (29.41)	211 (40.27)	95 (34.17)
Data available	2,903	544	668	1,087	746	203	221	524	278
**Age**									
mean ±SD, years	66.681 ± 0.35	66.029 ± 0.72	68.841 ± 0.95	66.499 ± 0.84	65.821 ± 0.64	67.078 ± 0.72	68.059 ± 0.30	68.611 ± 0.98	64.92± 10.72
Min-max	1–95	34–90	1– 92	31-92	20–95	43–89	34–95	31–91	12–87
Data available	2,341	480	539	877	543	159	202	427	248
**Year**									
2015	404 (11.99)	68 (11.00)	76 (9.93)	136 (10.45)	97 (11.81)	41 (16.14)	28 (10.33)	81 (13.39)	16 (5.35)
2016	651 (19.32)	117 (18.93)	121 (15.82)	296 (22.75)	125 (15.23)	59 (23.23)	61 (22.51)	131 (21.65)	37 (12.37)
2017	744 (22.08)	150 (24.27)	182 (23.79)	282 (21.68)	166 (20.22)	59 (23.23)	69 (25.46)	122 (20.17)	93 (31.10)
2018	902 (26.77)	147 (23.79)	200 (26.14)	315 (24.21)	298 (36.30)	45 (17.72)	50 (18.45)	180 (29.75)	76 (25.42)
2019	669 (19.85)	136 (22.01)	186 (24.31)	272 (20.91)	135 (16.44)	50 (19.69)	63 (23.25)	91 (15.04)	77 (25.75)
Data available	3,370	618	765	1,301	821	254	271	605	299
**Reported countries**									
United States	1,586 (47.06)	224 (36.25)	303 (39.61)	676 (51.96)	348 (42.39)	170 (66.93)	90 (33.21)	363 (60.00)	87 (29.10)
Great Britain	101 (3.00)	34 (5.50)	13 (1.70)	29 (2.23)	31 (3.78)	0 (0.00)	12 (4.43)	7 (1.16)	10 (3.34)
Canada	71 (2.11)	10 (1.62)	10 (1.31)	36 (2.77)	14 (1.71)	6 (2.36)	2 (0.74)	25 (4.13)	1 (0.33)
Germany	409 (12.14)	59 (9.55)	113 (14.77)	130 (9.99)	176 (21.44)	10 (3.94)	20 (7.38)	76 (12.56)	50 (16.72)
Japan	306 (9.08)	85 (13.75)	101 (13.20)	103 (7.92)	51 (6.21)	14 (5.51)	20 (7.38)	42 (6.94)	59 (19.73)
Italy	98 (2.91)	26 (4.21)	21 (2.75)	32 (2.46)	19 (2.31)	12 (4.72)	21 (7.75)	7 (1.16)	18(6.02)
Others	799 (23.71)	180 (29.13)	204 (26.67)	295 (22.67)	182 (22.17)	42 (16.54)	106 (39.11)	85 (14.05)	74 (24.75)
Data available	3,370	618	765	1,301	821	254	271	605	299
**Time to onset**									
Median, days	41	34	33	35	68	28	16	46.5	27
Quartile 1-3	9–114	9–125	8–69	8–90	21–139	4–113	4–85	8–114	8–93
Data available	1,502	326	347	519	443	61	125	254	185
**Outcome**									
Death	498 (16.35)	151 (25.12)	123 (17.45)	220 (19.56)	87 (10.71)	10 (5.71)	72 (26.67)	68 (13.93)	131 (44.11)
Life-threatening	273 (8.96)	44 (7.32)	63 (8.94)	78 (6.93)	97 (11.95)	16 (9.14)	45 (16.67)	31 (6.35)	19 (6.40)
Disability	55 (1.81)	4 (0.67)	7 (0.99)	25 (2.22)	25 (3.08)	1 (0.57)	0 (0.00)	14 (2.87)	0 (0.00)
Hospitalization	1,162 (38.15)	230 (38.27)	239 (33.90)	442 (39.29)	321 (39.53)	72 (41.14)	102 (37.78)	183 (37.50)	89 (29.97)
Congenital anomaly	0 (0.00)	0 (0.00)	0 (0.00)	0 (0.00)	0 (0.00)	0 (0.00)	0 (0.00)	0 (0.00)	0 (0.00)
Other serious	1,058 (34.73)	172 (28.62)	273 (38.72)	360 (32.00)	282 (34.73)	76 (43.43)	51 (18.89)	192 (39.34)	58 (19.53)
Required intervention	0 (0.00)	0 (0.00)	0 (0.00)	0 (0.00)	0 (0.00)	0 (0.00)	0 (0.00)	0 (0.00)	0 (0.00)
Data available	3,046	601	705	1,125	812	175	270	488	297

#### Time to Onset

**Figure 2:**
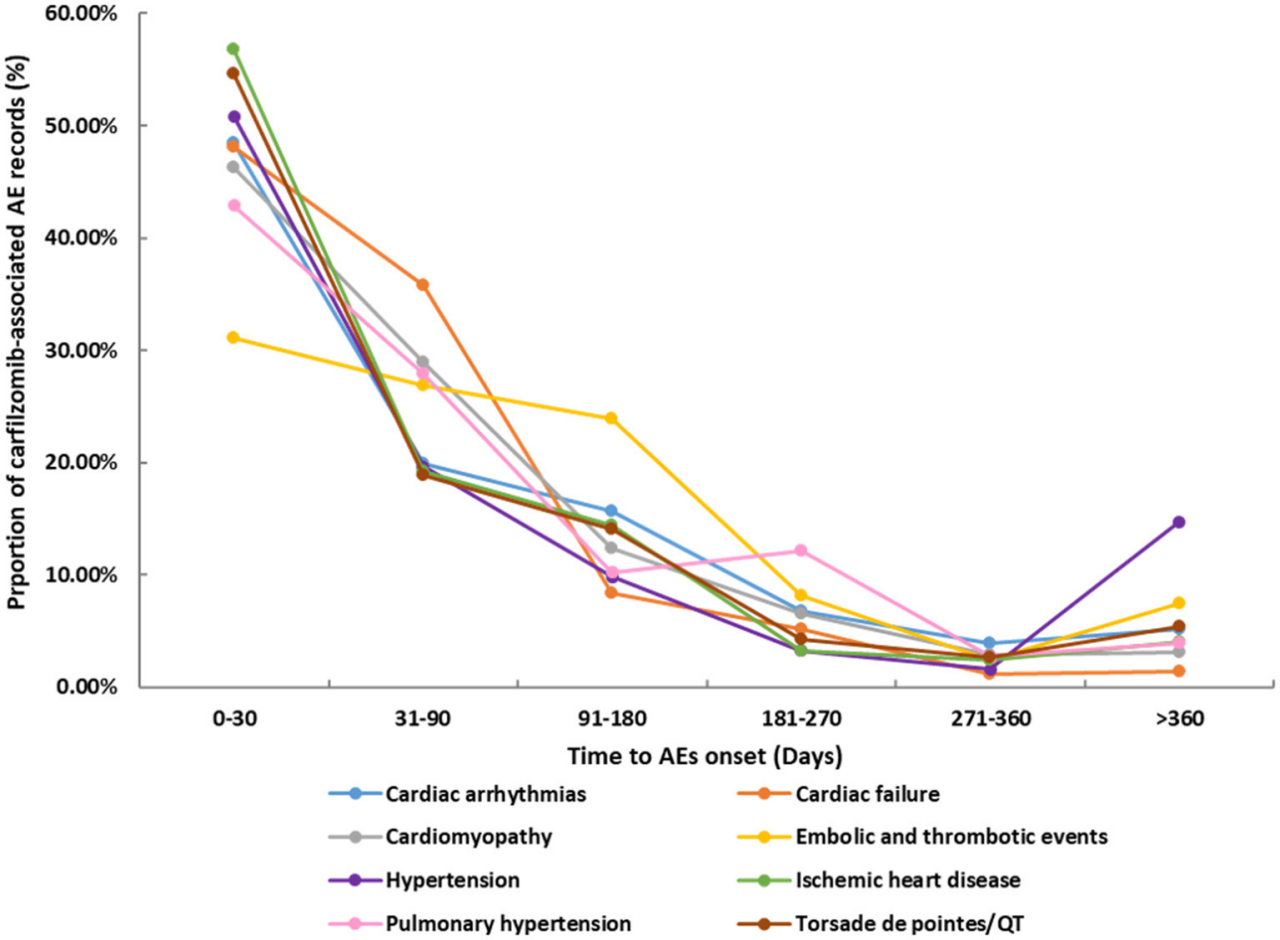
Time to onset for different categories of cardiovascular toxicities.

The differential spectra of time to onset with class-specific cardiovascular toxicities are presented in Figure 2. After exclusion of records with missing time (*n* = 1,868), overall, the median onset time of cardiovascular events was 41 days (Q1–Q3: 9–114 days) after carfilzomib initiation for all categories (Table 2). The cumulative proportions of cardiovascular records that occurred within the first 1 month and 3 months were 42.74 and 69.04%, respectively. Ischemic heart disease showed the shortest median time of 16 days (Q1–Q3: 4–85 days), followed by the 27 days (Q1–Q3: 8–93 days) of Torsade de pointes/QT prolongation. Embolic and thrombotic events had the longest median time of 68 days (Q1–Q3: 21–139 days), followed by the 46.5 days (Q1–Q3: 8–114 days) of pulmonary hypertension.

#### Outcome

**Figure 3:**
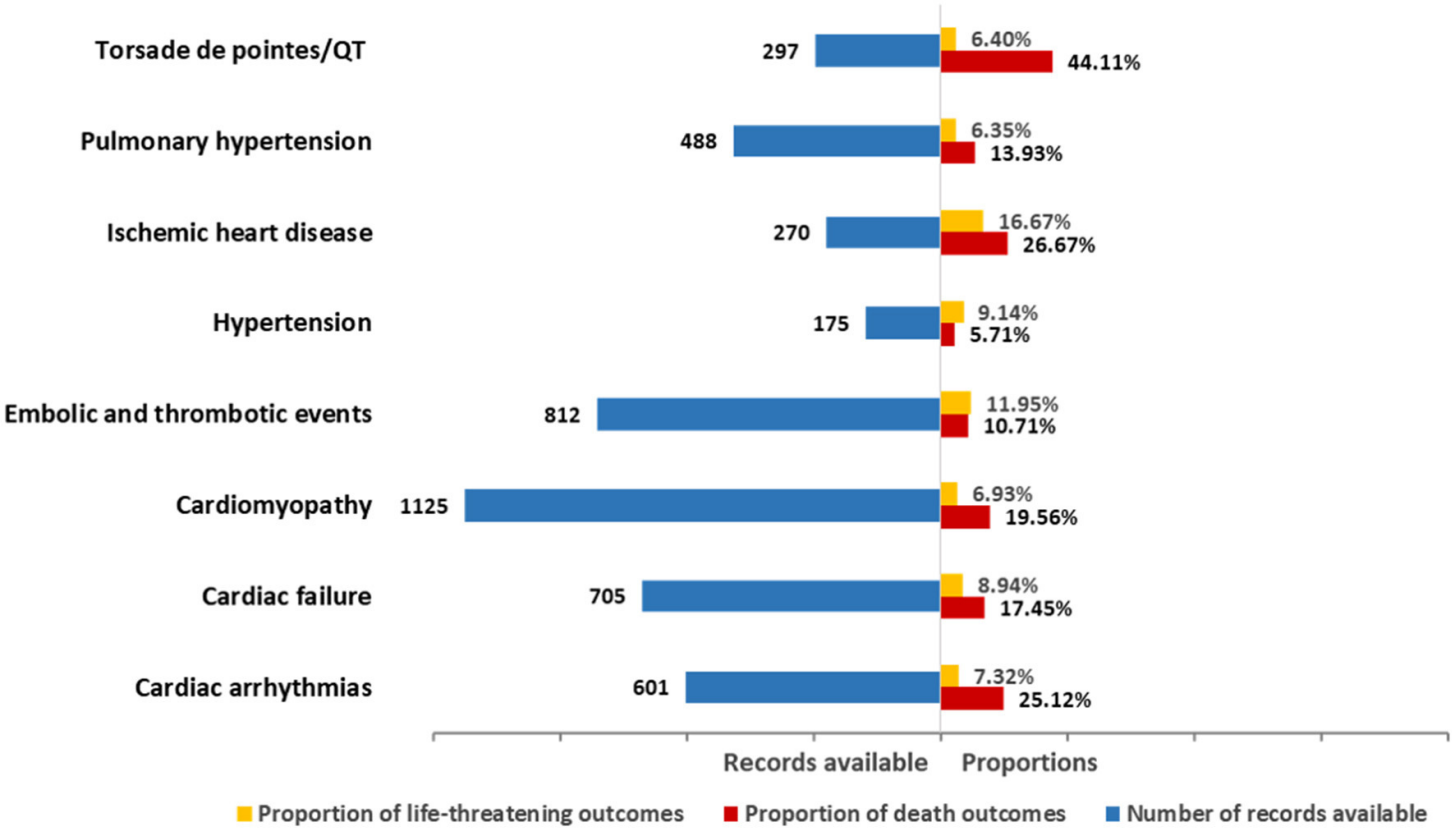
Records and death proportions in class-specific cardiovascular AE.

The death and life-threatening proportions according to types of cardiovascular toxicity are shown in Figure 3. In general, death accounted for 16.35% of all associated records with available final outcome information (Table 2). Upon further analysis, the severity of these events varied. Specifically, Torsade de pointes/QT prolongation was highlighted with 44.11% of records resulting in death. Ischemic heart disease showed the highest proportion (16.67%) of life-threatening conditions. Cardiac arrhythmias, cardiac failure, cardiomyopathy, embolic and thrombotic events, and pulmonary hypertension had death in 10–25% of the records (25.12, 17.45, 19.56, 10.71, and 13.93%, respectively). Hypertension presented the lowest proportion of death in the records.

### Disproportionality Analysis

**Figure 4:**
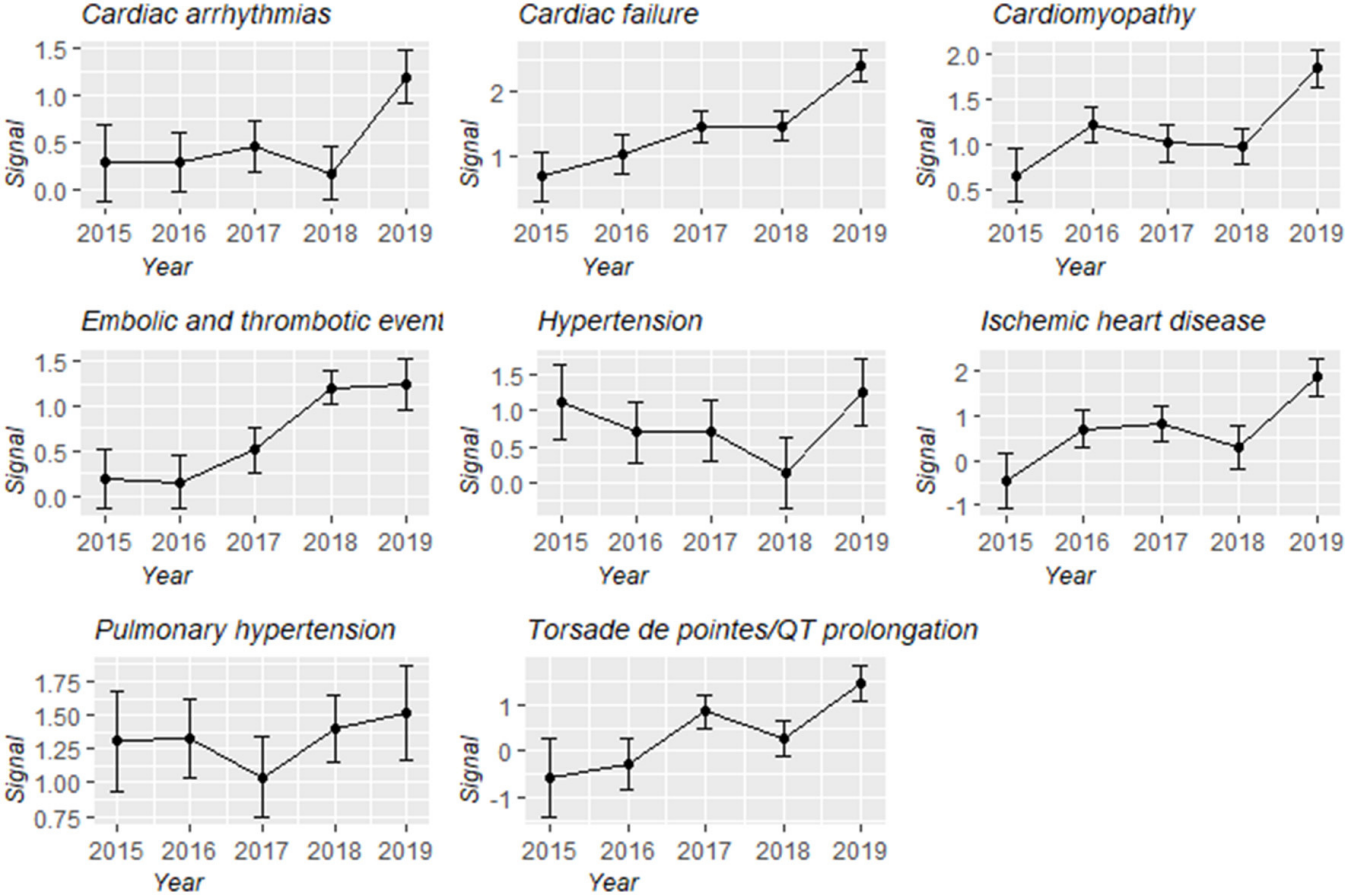
Information component and its 95% credibility interval over time for different types of carfilzomib-associated cardiovascular toxicities.

Overall, cardiovascular complications were over-reported in the people administrated with carfilzomib compared with the people in the entire database (IC_025_/ ROR_025_ = 0.85/1.95) (Table 3). Upon further analysis, all eight broad entities consistently maintained this trend. Cardiomyopathy-related complications (*N* = 1,301, 38.61%), embolic and thrombotic events (*N* = 821, 24.36%), and cardiac failure (*N* = 765, 22.70%) largely comprised the reported problems. Notably, the magnitude of disproportionality association was the highest for cardiac failure (IC_025_/ROR_025_ = 1.33/2.59), followed by pulmonary hypertension (IC_025_/ROR_025_ = 1.19/2.34). In addition, changes in IC values and their 95% confidence intervals over time for different types of cardiovascular events were characterized (Figure 4). Although there were distinctions existing across eight groups, an upward trend was observed and signal values all reached the peak at 2019.

**Table 3:** Results of the disproportionality analysis for cardiovascular records (total and specific) associated with carfilzomib^*^.

**Cardiovascular events**	***N***	**IC**	**IC_**025**_**	**IC_**975**_**	**ROR**	**ROR_**025**_**	**ROR_**975**_**
Total	3,370	0.90	0.85	0.96	2.02	1.95	2.10
Cardiac arrhythmias	618	0.47	0.34	0.60	1.40	1.29	1.51
Cardiac failure	765	1.45	1.33	1.57	2.79	2.59	3.00
Cardiomyopathy	1,301	1.15	1.06	1.24	2.29	2.17	2.43
Embolic and thrombotic events	821	0.71	0.60	0.83	1.67	1.55	1.79
Hypertension	254	0.75	0.54	0.95	1.69	1.49	1.91
Ischemic heart disease	271	0.63	0.43	0.83	1.55	1.38	1.75
Pulmonary hypertension	605	1.32	1.19	1.46	2.54	2.34	2.76
Torsade de pointes/QT prolongation	299	0.52	0.33	0.71	1.44	1.29	1.62

* *N, number of records; IC_025_, the lower limit of a 95% CI for the IC; IC_975_, the upper limit of a 95% CI; ROR_025_, the lower limit of the 95%CI of ROR; ROR_975_, the upper limit of the 95%CI of ROR. IC_025_0 _>_ and ROR_025_ >1 was deemed a signal*.

**Figure 5:**
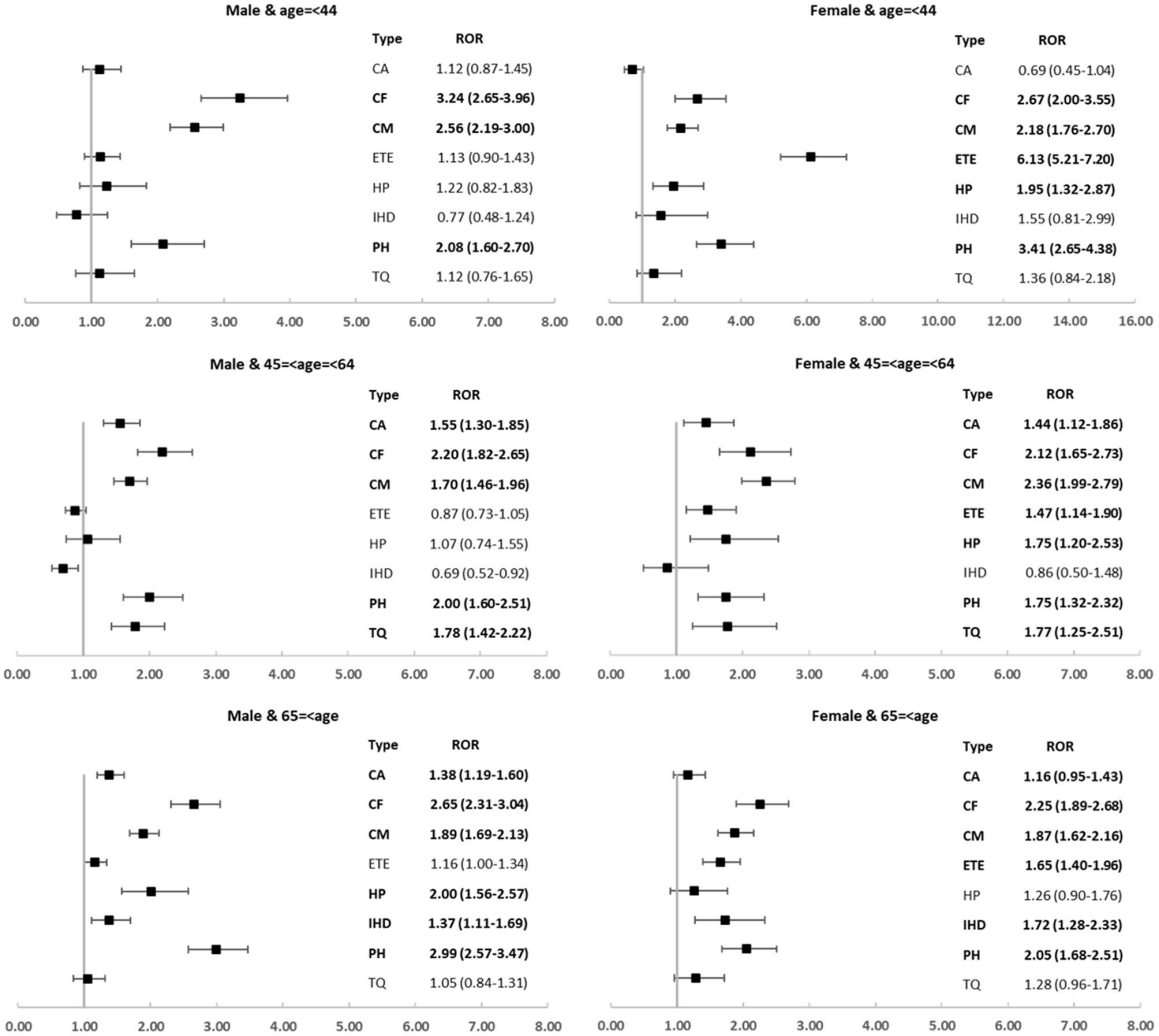
The spectrum of cardiovascular toxicity among different carfilzomib subpopulations.

Moreover, we explored the spectrum of cardiovascular toxicity among different carfilzomib subpopulations and revealed several disproportionate and intriguing associations. Cardiac failure, cardiomyopathy, and pulmonary hypertension were over-reported in all subgroups; by contrast, embolic and thrombotic events only disproportionately occurred in female groups and ischemic heart disease was detected as signal only in older subgroups (Figure 5).

## Discussion

Carfilzomib is an effective PI agent with an overall tolerable safety profile for the treatment of relapsed or refractory MM (20). The advent of this agent has contributed to significant survival improvements in this challenging population and it is now used with increasing frequency (21). However, there is growing evidence that carfilzomib use is linked with higher than the expected rate of cardiovascular risks. Since being approved in 2012, there has been a large body of articles reporting cardiovascular complications associated with this agent, encompassing cardiac failure, cardiomyopathy, hypertension, cardiac arrhythmias, ischemic heart disease, etc., (21–23). However, details of these cardiovascular events among the general population remain unclear. There is growing recognition of the need to provide a comprehensive understanding of the cardiovascular toxicity profile after carfilzomib administration from real-world evidence.

To our knowledge, the current study presents the most extensive and exhaustive characterization of carfilzomib-associated cardiovascular toxicities based on the FAERS database. It is a detailed and important examination of the currently available observational data, with the potential to improve management in affected patients. A large number of records were involved, enabling us to conduct a systematic analysis of the frequency, spectrum, clinical features, timing, and outcomes of carfilzomib-related cardiovascular toxicities. We have enumerated the notable and interesting findings as below:

First, cardiovascular records accounted for a proportion of 14.84% of all carfilzomib-associated records, which was similar to the incidence rate (18.1%) reported in an extensive meta-analysis (8). Importantly, our study suggested that carfilzomib use was associated with a significantly increased risk of cardiovascular AEs, which was evident by the higher disproportionality in contrast with the whole database. Since carfilzomib is mostly prescribed for MM and it has been reported that many patients with MM have cardiovascular comorbidities or relevant risk factors (24, 25). Therefore, we conducted an additional disproportionality analysis in this subpopulation. Results are shown in Table S9. In general, despite being undermined, the disproportionality association between cardiovascular events and carfilzomib administration remains significant (IC_025_/ ROR_025_ = 0.46/1.46). There are several published studies supporting that augmented cardiovascular risk might be associated with carfilzomib utilization. A large meta-analysis has reported that carfilzomib utilization significantly increased the risk of cardiotoxicity and arterial hypertension (26). Similarly, a study pooling data across phase 1–3 trials with 2000 patients exposed to carfilzomib demonstrated that patients receiving carfilzomib had an increased incidence of cardiovascular events (27). Subsequently, Waxman et al. performed the first meta-analysis investigating the potential association of carfilzomib with cardiovascular AEs, concluding that carfilzomib was associated with an augmented risk of high-grade and all-grade cardiovascular events compared with noncarfilzomib-receiving controls (8). Currently, the precise pathogenesis of cardiovascular events in carfilzomib receivers has not been completely understood, though several underlying mechanisms included endothelial effects, proteasome inhibition, and cardiorenal syndrome were proposed (28, 29). More mechanistic studies are needed in the future to unravel the mechanism of the carfilzomib-associated cardiovascular toxicity.

Second, we noticed that the eight broad entities of cardiovascular toxicity were all disproportionately associated with carfilzomib when data mining in the entire database, ranging from cardiac arrhythmias to cardiac failure. When further exploring these toxicities in the MM-related indication subset, only signal of embolic and thrombotic events disappeared, while others were detected as disproportionality signals in line with our main analysis (Table S9). Taken together, it indicated that carfilzomib-associated cardiovascular events were highly versatile, which was largely consistent with those being reported in the two extensive reviews (29, 30). Cardiomyopathy-related complications were the most common reported cardiovascular AEs in our study regardless of the use of the whole dataset or the MM-related indication subset, which is not in accordance with previous literature that suggested hypertension (31, 32) was the most prevalent. In our main analysis, cardiac failure occurred in 3.37% of all carfilzomib-associated records, approximately to 4.1% in Waxman et al.'s study (8), which is highlighted with the strongest disproportionality. Notably, a general increasing trend of the signal was captured across all categories over time using the entire database, which needs to be paid attention. With further data mining in different subpopulations, cardiac failure was over-reported in all subgroups. Data from a recent meta-analysis included four phase III RCTs also supports the elevated risk of cardiac failure secondary to carfilzomib usage (32).

Third, we observed that the spectra of cardiovascular toxicities differed in subpopulations. It is worthwhile to mention that several carfilzomib-associated cardiovascular complications tend to occur in specific populations. We found that women were disproportionately affected by embolic and thrombotic events, regardless of the age; by contrast, older patients were disproportionately affected by ischemic heart disease, regardless of sex. These findings suggested that age and sex might be important biological variables and deserve more attention. Continued efforts are needed concerning the identification and quantification of the sex and age differences, as well as to explore the underlying mechanisms and to monitor and manage the affected patients more efficiently.

Fourth, despite several studies have noted that carfilzomib-associated toxicities occurred early (usually within two cycles), the time-to-onset of these events after administrating carfilzomib is still unclear (3, 6, 33). Our study partially fill the knowledge gap and provide useful information for future studies. Note worthily, we found that cardiovascular toxicities tended to occur early after giving carfilzomib, with a median onset time of 41 days and majority of events occurring within 3 months. Importantly, we described the differential spectra of time-to-onset according to different types of cardiovascular AEs. We observed that median time to onset occurred fairly early for ischemic heart disease (16 days, Q1–Q3: 4–85 days), and was the most delayed for embolic thrombotic events (68 days, Q1–Q3: 21–139 days). It is worthwhile to recognize the difference of time-to-onset between different types of cardiovascular toxicities; furthermore, our findings underscore a need for early and closely follow-up after carfilzomib treatment, particularly in the first 3 months, to ensure an early intervention in the affected population.

Fifth, we noticed that death accounted for 16.35% of all associated records, implying the significant impact that cardiovascular complications imposed on the patient mortality. Additionally, we evaluated and compared outcomes according to different types of cardiovascular events. It turned out that the severity of these events varied in categories. Given the potential hyperacute onset of these diseases, early and intensified monitoring is particularly necessary. It has been recommended to hold carfilzomib until toxicity reducing to grade <2 when cardiovascular AEs occur, and if the decision is made to re-challenge the patients, a dose reduction may be considered (33). Animal model studies have noted several new agents to potentially attenuate and counteract carfilzomib-induced cardiovascular toxicity (34, 35); nevertheless, whether these agents are useful in clinical practice needs further investigation.

Last but not least, in our study, Torsade de pointes/QT prolongation was identified as a new signal linked to carfilzomib administration (IC_025_/ROR_025_ = 0.33/1.29) and was particularly notable with the highest death proportion (44.11%). Torsades de pointes is a specific form of ventricular arrhythmia associated with prolongation of the corrected QT interval and very frequently due to drug administration (36). It is rare but constitute a significant problem because of sudden cardiac death. Despite there have been several published studies regarding the cardiovascular toxicities after carfilzomib, this agent is not known to cause Torsade de pointes/QT prolongation according to previous studies. We postulated that this may be related to the rarity and high mortality of this toxicity. Compared with existing studies, the strength of enormous records at a national level supports our study to explore several rare cardiovascular events and quantify all possible signals. Our study might supplement existing literatures by revealing this novel potential cardiovascular toxicity following carfilzomib use. We hope our work can inspire future studies to gain a deeper insight into this newly identified signal.

The major strength of the presented study is that enormous records involved at a national level from the real-world setting, which enables a better evaluation of risk AEs and broader generalization of our findings. However, several limitations in the study should also be acknowledged. First, there is an inevitable and unquantifiable bias intrinsic to the use of spontaneous data, such as over-reporting, under-reporting, and incomplete information. Second, FAERS does not provide detail clinical information regarding the severity of AEs which might contribute to a better comprehensive analysis. Third, most of the reporting data in FAERS database come from America and Europe countries. Apart from Japan, other Asian countries have very few records in the database compared with America and Europe countries, which would lead to geographic bias of the results. Forth, the exact number of patients exposed to carfilzomib in the FAERS cannot be evaluated, therefore, we are unable to determine AE incidence. Fifth, changing awareness of toxicities over time could affect reporting behaviors (17). Sixth, despite disproportionality methods are efficient in signal detection, their shortcomings in dealing with confounding like masking effect and co-prescription should also be recognized (37). It is important to note that the significant disproportionality in this study did not support a causal relationship; instead, it indicates a potential safety signal which should be followed up and validated by prospective studies.

## Conclusion

The exhaustive pharmacovigilance analysis contributes to the cumulative knowledge about the cardiovascular safety of carfilzomib. Several toxicities are highlighted and deserve further attention, such as cardiac failure with the strongest disproportionality association, ischemic heart disease with fairly early onset time and the highest proportion of life-threatening conditions, and Torsade de pointes/QT prolongation with the highest death proportion.

## Data Availability Statement

Publicly available datasets were analyzed in this study. This data can be found here: https://fis.fda.gov/extensions/FPD-QDE-FAERS/FPD-QDE-FAERS.html.

## Author Contributions

YZ, XY, and FH: conception and design. JH: administrative support. JX, XG, and ZL: collection and assembly of data. YZ and FH: data analysis and interpretation. YZ: manuscript writing. XY, FH, YC, ZG, and XZ: manuscript reviewing and revising. All authors approved the final manuscript.

## Funding

This study was supported by the National Nature Science Foundation of China (No. 82073671), the Leading Talents of Public Health in Shanghai (No. GWV-10.2-XD22), the Shanghai Municipal Commission of Health and Family Planning Fund for Excellent Young Scholars (No. 2018YQ47), and the Excellent Young Scholars of public health in Shanghai (No. GWV-10.2-YQ33), three-year Action Program of Shanghai Municipality for Strengthening the Construction of Public Health System (GWV-10.1-XK05) Big Data and Artificial Intelligence Application, and Military Key Discipline Construction Project (Health Service–Naval Health Service Organization and Command) (No.03).

## Conflict of Interest

The authors declare that the research was conducted in the absence of any commercial or financial relationships that could be construed as a potential conflict of interest.

## Publisher's Note

All claims expressed in this article are solely those of the authors and do not necessarily represent those of their affiliated organizations, or those of the publisher, the editors and the reviewers. Any product that may be evaluated in this article, or claim that may be made by its manufacturer, is not guaranteed or endorsed by the publisher.

## Abbreviations

AE: adverse event
FAERS: FDA adverse event reporting system
HLGT: high-level group term
HLT: high-level term
IC: information component
MedDRA: Medical Dictionary for Regulatory Activities
MM: multiple myeloma
N: number of records
PI: proteasome inhibitor
PT: preferred term
ROR: reporting odds ratio
SMQs: Standardized MedDRA Queries
SOC: system organ class
U.S.: United States.
