# Supplementary material for: Cardiovascular Toxicity of Carfilzomib: The Real-World Evidence Based on the Adverse Event Reporting System Database of the FDA, the United States
Source: Front Cardiovasc Med. 2021 Sep 27;8:735466. doi: 10.3389/fcvm.2021.735466 (PMC8503541; doi:10.3389/fcvm.2021.735466)
Supplement: Supplementary file 1 [file Data_Sheet_1.docx]

**Table S1****.** SMQ “Cardiac arrhythmias” and PTs used according to MedDRA 22.0

| SMQ: Cardiac arrhythmias | |
| --- | --- |
| Chronotropic incompetence | Sinus node dysfunction |
| Electrocardiogram repolarisation abnormality | Wandering pacemaker |
| Electrocardiogram RR interval prolonged | Arrhythmia |
| Electrocardiogram U wave inversion | Heart alternation |
| Electrocardiogram U wave present | Heart rate irregular |
| Electrocardiogram U-wave abnormality | Pacemaker generated arrhythmia |
| Sudden cardiac death | Pacemaker syndrome |
| Bezold-Jarisch reflex | Paroxysmal arrhythmia |
| Bradycardia | Pulseless electrical activity |
| Cardiac arrest | Reperfusion arrhythmia |
| Cardiac death | Withdrawal arrhythmia |
| Cardiac telemetry abnormal | Arrhythmia supraventricular |
| Cardio-respiratory arrest | Atrial fibrillation |
| Central bradycardia | Atrial flutter |
| Electrocardiogram abnormal | Atrial parasystole |
| Electrocardiogram ambulatory abnormal | Atrial tachycardia |
| Electrocardiogram change | Congenital supraventricular tachycardia |
| Heart rate abnormal | Frederick's syndrome |
| Heart rate decreased | Junctional ectopic tachycardia |
| Heart rate increased | Sinus tachycardia |
| Loss of consciousness | Supraventricular extrasystoles |
| Palpitations | Supraventricular tachyarrhythmia |
| Rebound tachycardia | Supraventricular tachycardia |
| Respiratory sinus arrhythmia magnitude abnormal | ECG P wave inverted |
| Respiratory sinus arrhythmia magnitude decreased | Electrocardiogram P wave abnormal |
| Respiratory sinus arrhythmia magnitude increased | Retrograde p-waves |
| Sudden death | Anomalous atrioventricular excitation |
| Syncope | Cardiac fibrillation |
| Tachycardia | Cardiac flutter |
| Tachycardia paroxysmal | Extrasystoles |
| Bradyarrhythmia | Tachyarrhythmia |
| Ventricular asystole | Accelerated idioventricular rhythm |
| Accessory cardiac pathway | Cardiac fibrillation |
| Adams-Stokes syndrome | Parasystole |
| Agonal rhythm | Rhythm idioventricular |
| Atrial conduction time prolongation | Torsade de pointes |
| Atrioventricular block | Ventricular arrhythmia |
| Atrioventricular block complete | Ventricular extrasystoles |
| Atrioventricular block first degree | Ventricular fibrillation |
| Atrioventricular block second degree | Ventricular flutter |
| Atrioventricular conduction time shortened | Ventricular parasystole |
| Atrioventricular dissociation | Ventricular pre-excitation |
| Bifascicular block | Ventricular tachyarrhythmia |
| Brugada syndrome | Ventricular tachycardia |
| Bundle branch block | Arrhythmia neonatal |
| Bundle branch block bilateral | Arrhythmogenic right ventricular dysplasia |
| Bundle branch block left | Atrioventricular node dispersion |
| Bundle branch block right | Brugada syndrome |
| Conduction disorder | Foetal arrhythmia |
| Defect conduction intraventricular | Foetal heart rate disorder |
| Electrocardiogram delta waves abnormal | Foetal tachyarrhythmia |
| Electrocardiogram PQ interval prolonged | Heart block congenital |
| Electrocardiogram PQ interval shortened | Junctional ectopic tachycardia |
| Electrocardiogram PR prolongation | Long QT syndrome congenital |
| Electrocardiogram PR shortened | Lown-Ganong-Levine syndrome |
| Electrocardiogram QRS complex prolonged | Neonatal bradyarrhythmia |
| Electrocardiogram QT prolonged | Neonatal tachyarrhythmia |
| Electrocardiogram repolarisation abnormality | Wolff-Parkinson-White syndrome congenital |
| Lenegre's disease | Baseline foetal heart rate variability disorder |
| Long QT syndrome | Bradycardia foetal |
| Paroxysmal atrioventricular block | Bradycardia neonatal |
| Sinoatrial block | Cardiac arrest neonatal |
| Trifascicular block | Cardio-respiratory arrest neonatal |
| Ventricular dyssynchrony | Foetal heart rate acceleration abnormality |
| Wolff-Parkinson-White syndrome | Foetal heart rate deceleration abnormality |
| Nodal arrhythmia | Neonatal sinus bradycardia |
| Nodal rhythm | Neonatal sinus tachycardia |
| Sinus arrest | Neonatal tachycardia |
| Sinus arrhythmia | Nonreassuring foetal heart rate pattern |
| Sinus bradycardia | Tachycardia foetal |

**Table S2.** SMQ “Cardiac failure” and PTs used according to MedDRA 22.0

| SMQ: Cardiac failure | |
| --- | --- |
| Acute left ventricular failure | Cardio-respiratory distress |
| Acute pulmonary oedema | Cardiothoracic ratio increased |
| Acute right ventricular failure | Central venous pressure increased |
| Cardiac asthma | Diastolic dysfunction |
| Cardiac failure | Dilatation ventricular |
| Cardiac failure acute | Dyspnoea paroxysmal nocturnal |
| Cardiac failure chronic | Heart transplant |
| Cardiac failure congestive | Hepatic vein dilatation |
| Cardiac failure high output | Implantable cardiac monitor replacement |
| Cardiogenic shock | Intracardiac pressure increased |
| Cardiohepatic syndrome | Jugular vein distension |
| Cardiopulmonary failure | Left ventricular diastolic collapse |
| Cardiorenal syndrome | Left ventricular dilatation |
| Chronic left ventricular failure | Left ventricular dysfunction |
| Chronic right ventricular failure | Left ventricular enlargement |
| Cor pulmonale | Lower respiratory tract congestion |
| Cor pulmonale acute | Myocardial depression |
| Cor pulmonale chronic | Nocturnal dyspnoea |
| Ejection fraction decreased | N-terminal prohormone brain natriuretic peptide abnormal |
| Hepatic congestion | N-terminal prohormone brain natriuretic peptide increased |
| Hepatojugular reflux | Oedema |
| Left ventricular failure | Oedema blister |
| Low cardiac output syndrome | Oedema due to cardiac disease |
| Neonatal cardiac failure | Oedema neonatal |
| Obstructive shock | Oedema peripheral |
| Pulmonary oedema | Orthopnoea |
| Pulmonary oedema neonatal | Peripheral oedema neonatal |
| Radiation associated cardiac failure | Peripheral swelling |
| Right ventricular ejection fraction decreased | Post cardiac arrest syndrome |
| Right ventricular failure | Prohormone brain natriuretic peptide abnormal |
| Ventricular failure | Prohormone brain natriuretic peptide increased |
| Artificial heart implant | Pulmonary congestion |
| Atrial natriuretic peptide abnormal | Right ventricular diastolic collapse |
| Atrial natriuretic peptide increased | Right ventricular dilatation |
| Bendopnoea | Right ventricular dysfunction |
| Brain natriuretic peptide abnormal | Right ventricular enlargement |
| Brain natriuretic peptide increased | Scan myocardial perfusion abnormal |
| Cardiac cirrhosis | Stroke volume decreased |
| Cardiac contractility modulation therapy | Surgical ventricular restoration |
| Cardiac device reprogramming | Systolic dysfunction |
| Cardiac dysfunction | Venous pressure increased |
| Cardiac index decreased | Venous pressure jugular abnormal |
| Cardiac output decreased | Venous pressure jugular increased |
| Cardiac resynchronisation therapy | Ventricular assist device insertion |
| Cardiac ventriculogram abnormal | Ventricular compliance decreased |
| Cardiac ventriculogram left abnormal | Ventricular dysfunction |
| Cardiac ventriculogram right abnormal | Ventricular dyssynchrony |
| Cardiomegaly | Wall motion score index abnormal |

**Table** **S3.** SMQ “Cardiomyopathy” and PTs used according to MedDRA 22.0

| SMQ: Cardiomyopathy | |
| --- | --- |
| Atrial septal defect acquired | Decreased ventricular preload |
| Biopsy heart abnormal | Diastolic dysfunction |
| Cardiac amyloidosis | Dilatation atrial |
| Cardiac hypertrophy | Dilatation ventricular |
| Cardiac iron overload | Directional Doppler flow tests abnormal |
| Cardiac sarcoidosis | Dyspnoea |
| Cardiac septal hypertrophy | ECG signs of ventricular hypertrophy |
| Cardiomyopathy | Echocardiogram abnormal |
| Cardiomyopathy acute | Electrocardiogram abnormal |
| Cardiomyopathy alcoholic | Electrocardiogram change |
| Cardiomyopathy neonatal | Electrocardiogram PR segment depression |
| Cardiotoxicity | Electrocardiogram U wave inversion |
| Chagas' cardiomyopathy | Endocardial fibroelastosis |
| Congestive cardiomyopathy | External counterpulsation |
| Cytotoxic cardiomyopathy | Gonococcal heart disease |
| Diabetic cardiomyopathy | Heart and lung transplant |
| Ejection fraction abnormal | Heart transplant |
| Ejection fraction decreased | Hepatomegaly |
| Eosinophilic myocarditis | Hyperdynamic left ventricle |
| HIV cardiomyopathy | Hypersensitivity myocarditis |
| Hypertensive cardiomyopathy | Implantable cardiac monitor replacement |
| Hypertrophic cardiomyopathy | Increased ventricular preload |
| Ischaemic cardiomyopathy | Intracardiac pressure increased |
| Metabolic cardiomyopathy | Irregular breathing |
| Myocardial calcification | Labile blood pressure |
| Myocardial fibrosis | Left atrial dilatation |
| Myocardial haemorrhage | Left atrial enlargement |
| Non-obstructive cardiomyopathy | Left atrial volume abnormal |
| Obesity cardiomyopathy | Left atrial volume decreased |
| Peripartum cardiomyopathy | Left atrial volume increased |
| Pulmonary arterial wedge pressure increased | Left ventricular dilatation |
| Restrictive cardiomyopathy | Left ventricular dysfunction |
| Right ventricular ejection fraction decreased | Left ventricular end-diastolic pressure decreased |
| Stress cardiomyopathy | Left ventricular enlargement |
| Tachycardia induced cardiomyopathy | Left ventricular failure |
| Thyrotoxic cardiomyopathy | Left ventricular heave |
| Ventricular septal defect acquired | Lupus myocarditis |
| Viral cardiomyopathy | Lyme carditis |
| Abnormal precordial movement | Malarial myocarditis |
| Acquired cardiac septal defect | Mental status changes |
| Acute left ventricular failure | Multiple gated acquisition scan abnormal |
| Alcohol septal ablation | Myocardiac abscess |
| Arrhythmia | Myocardial necrosis marker increased |
| Arrhythmia supraventricular | Myocarditis |
| Artificial heart implant | Myocarditis bacterial |
| Ascites | Myocarditis helminthic |
| Atrial enlargement | Myocarditis infectious |
| Atrial hypertrophy | Myocarditis meningococcal |
| Atrial pressure increased | Myocarditis mycotic |
| Autoimmune myocarditis | Myocarditis post infection |
| Bendopnoea | Myocarditis septic |
| Blood pressure diastolic abnormal | Myocarditis syphilitic |
| Blood pressure diastolic decreased | Myocarditis toxoplasmal |
| Blood pressure diastolic increased | Myoglobinaemia |
| Blood pressure fluctuation | Myoglobinuria |
| Blood pressure inadequately controlled | Nocturia |
| Blood pressure systolic abnormal | Nuclear magnetic resonance imaging thoracic abnormal |
| Blood pressure systolic decreased | Oedema |
| Blood pressure systolic increased | Orthostatic hypotension |
| Cardiac aneurysm | Palpitations |
| Cardiac arrest | Papillary muscle disorder |
| Cardiac contractility modulation therapy | Papillary muscle haemorrhage |
| Cardiac device reprogramming | Radiation myocarditis |
| Cardiac dysfunction | Right atrial dilatation |
| Cardiac electrophysiologic study abnormal | Right atrial enlargement |
| Cardiac failure | Right atrial pressure increased |
| Cardiac failure acute | Right ventricle outflow tract obstruction |
| Cardiac failure chronic | Right ventricular dilatation |
| Cardiac failure congestive | Right ventricular enlargement |
| Cardiac function test abnormal | Right ventricular heave |
| Cardiac imaging procedure abnormal | Right ventricular systolic pressure decreased |
| Cardiac index abnormal | Scan myocardial perfusion abnormal |
| Cardiac index decreased | Sudden cardiac death |
| Cardiac index increased | Sudden death |
| Cardiac monitoring abnormal | Surgical ventricular restoration |
| Cardiac operation | Syncope |
| Cardiac output decreased | Systolic anterior motion of mitral valve |
| Cardiac pseudoaneurysm | Systolic dysfunction |
| Cardiac resynchronisation therapy | Ultrasound Doppler abnormal |
| Cardiac ventricular scarring | Vascular resistance pulmonary increased |
| Cardiac ventriculogram abnormal | Ventricular arrhythmia |
| Cardiac ventriculogram left abnormal | Ventricular assist device insertion |
| Cardiac ventriculogram right abnormal | Ventricular dysfunction |
| Cardiomegaly | Ventricular dyskinesia |
| Cardiothoracic ratio increased | Ventricular dyssynchrony |
| Cardiovascular disorder | Ventricular enlargement |
| Cardiovascular function test abnormal | Ventricular hyperkinesia |
| Chest pain | Ventricular hypertrophy |
| Chest X-ray abnormal | Ventricular hypokinesia |
| Computerised tomogram thorax abnormal | Ventricular remodelling |
| Coxsackie carditis | Viral myocarditis |
| Coxsackie myocarditis | Wall motion score index abnormal |
| Cytomegalovirus myocarditis |  |

**Table S4.** SMQ “Embolic and thrombotic events” and PTs used according to MedDRA 22.0

| SMQ: Embolic and thrombotic events | |
| --- | --- |
| Acute aortic syndrome | Pulmonary vein occlusion |
| Acute myocardial infarction | Pulmonary veno-occlusive disease |
| Amaurosis | Pulmonary venous thrombosis |
| Amaurosis fugax | Renal vein embolism |
| Angioplasty | Renal vein occlusion |
| Aortic bypass | Renal vein thrombosis |
| Aortic embolus | Retinal vein occlusion |
| Aortic surgery | Retinal vein thrombosis |
| Aortic thrombosis | SI QIII TIII pattern |
| Aortogram abnormal | Splenic vein occlusion |
| Arterectomy | Splenic vein thrombosis |
| Arterectomy with graft replacement | Subclavian vein occlusion |
| Arterial angioplasty | Subclavian vein thrombosis |
| Arterial bypass occlusion | Superior sagittal sinus thrombosis |
| Arterial bypass operation | Superior vena cava occlusion |
| Arterial bypass thrombosis | Superior vena cava syndrome |
| Arterial graft | Thrombophlebitis |
| Arterial occlusive disease | Thrombophlebitis migrans |
| Arterial stent insertion | Thrombophlebitis neonatal |
| Arterial therapeutic procedure | Thrombophlebitis superficial |
| Arterial thrombosis | Thrombosed varicose vein |
| Arteriogram abnormal | Thrombosis corpora cavernosa |
| Arteriogram carotid abnormal | Transverse sinus thrombosis |
| Arteriotomy | Vena cava embolism |
| Atherectomy | Vena cava filter insertion |
| Atherosclerotic plaque rupture | Vena cava filter removal |
| Atrial appendage closure | Vena cava thrombosis |
| Atrial appendage resection | Venogram abnormal |
| Basal ganglia infarction | Venoocclusive disease |
| Basilar artery occlusion | Venoocclusive liver disease |
| Basilar artery thrombosis | Venous angioplasty |
| Blindness transient | Venous occlusion |
| Brachiocephalic artery occlusion | Venous operation |
| Capsular warning syndrome | Venous recanalisation |
| Carotid angioplasty | Venous repair |
| Carotid arterial embolus | Venous stent insertion |
| Carotid artery bypass | Venous thrombosis |
| Carotid artery occlusion | Venous thrombosis in pregnancy |
| Carotid artery stent insertion | Venous thrombosis limb |
| Carotid artery thrombosis | Venous thrombosis neonatal |
| Carotid endarterectomy | Visceral venous thrombosis |
| Cerebellar artery occlusion | Administration site thrombosis |
| Cerebellar artery thrombosis | Adrenal thrombosis |
| Cerebral artery embolism | Angiogram abnormal |
| Cerebral artery occlusion | Angiogram cerebral abnormal |
| Cerebral artery stent insertion | Angiogram peripheral abnormal |
| Cerebral artery thrombosis | Antiphospholipid syndrome |
| Cerebral hypoperfusion | Application site thrombosis |
| Cerebrovascular insufficiency | Arteriovenous fistula occlusion |
| Cerebrovascular stenosis | Arteriovenous fistula thrombosis |
| Coeliac artery occlusion | Arteriovenous graft thrombosis |
| Coronary angioplasty | Artificial blood vessel occlusion |
| Coronary arterial stent insertion | Atrial thrombosis |
| Coronary artery bypass | Basal ganglia stroke |
| Coronary artery embolism | Bone infarction |
| Coronary artery occlusion | Brain stem embolism |
| Coronary artery reocclusion | Brain stem infarction |
| Coronary artery surgery | Brain stem stroke |
| Coronary artery thrombosis | Brain stem thrombosis |
| Coronary endarterectomy | Cardiac ventricular thrombosis |
| Coronary revascularisation | Catheter site thrombosis |
| Coronary vascular graft occlusion | Cerebellar embolism |
| Embolia cutis medicamentosa | Cerebellar infarction |
| Embolism arterial | Cerebral congestion |
| Endarterectomy | Cerebral infarction |
| Femoral artery embolism | Cerebral infarction foetal |
| Hepatic artery embolism | Cerebral ischaemia |
| Hepatic artery occlusion | Cerebral microembolism |
| Hepatic artery thrombosis | Cerebral septic infarct |
| Hypothenar hammer syndrome | Cerebral thrombosis |
| Iliac artery embolism | Cerebral vascular occlusion |
| Iliac artery occlusion | Cerebrospinal thrombotic tamponade |
| Intra-aortic balloon placement | Cerebrovascular accident |
| Intraoperative cerebral artery occlusion | Cerebrovascular accident prophylaxis |
| Ischaemic cerebral infarction | Cerebrovascular disorder |
| Ischaemic stroke | Cerebrovascular operation |
| Lacunar infarction | Choroidal infarction |
| Leriche syndrome | Collateral circulation |
| Mesenteric arterial occlusion | Coronary bypass thrombosis |
| Mesenteric arteriosclerosis | Device embolisation |
| Mesenteric artery embolism | Device occlusion |
| Mesenteric artery stenosis | Device related thrombosis |
| Mesenteric artery stent insertion | Diplegia |
| Mesenteric artery thrombosis | Directional Doppler flow tests abnormal |
| Myocardial infarction | Disseminated intravascular coagulation |
| Myocardial necrosis | Disseminated intravascular coagulation in newborn |
| Ophthalmic artery thrombosis | Embolic cerebral infarction |
| Papillary muscle infarction | Embolic pneumonia |
| Penile artery occlusion | Embolic stroke |
| Percutaneous coronary intervention | Embolism |
| Peripheral arterial occlusive disease | Foetal cerebrovascular disorder |
| Peripheral arterial reocclusion | Graft thrombosis |
| Peripheral artery angioplasty | Haemorrhagic adrenal infarction |
| Peripheral artery bypass | Haemorrhagic cerebral infarction |
| Peripheral artery occlusion | Haemorrhagic infarction |
| Peripheral artery stent insertion | Haemorrhagic stroke |
| Peripheral artery surgery | Haemorrhagic transformation stroke |
| Peripheral artery thrombosis | Haemorrhoids thrombosed |
| Peripheral embolism | Hemiparesis |
| Peripheral endarterectomy | Hemiplegia |
| Popliteal artery entrapment syndrome | Heparin-induced thrombocytopenia |
| Post procedural myocardial infarction | Hepatic infarction |
| Postinfarction angina | Hepatic vascular thrombosis |
| Precerebral artery occlusion | Implant site thrombosis |
| Precerebral artery thrombosis | Incision site vessel occlusion |
| Profundaplasty | Infarction |
| Pulmonary artery occlusion | Infusion site thrombosis |
| Pulmonary artery therapeutic procedure | Injection site thrombosis |
| Pulmonary artery thrombosis | Inner ear infarction |
| Pulmonary endarterectomy | Instillation site thrombosis |
| Pulmonary tumour thrombotic microangiopathy | Intestinal infarction |
| Renal artery angioplasty | Intracardiac mass |
| Renal artery occlusion | Intracardiac thrombus |
| Renal artery thrombosis | Medical device site thrombosis |
| Renal embolism | Mesenteric vascular insufficiency |
| Retinal artery embolism | Mesenteric vascular occlusion |
| Retinal artery occlusion | Microembolism |
| Retinal artery thrombosis | Monoparesis |
| Silent myocardial infarction | Monoplegia |
| Spinal artery embolism | Optic nerve infarction |
| Spinal artery thrombosis | Pancreatic infarction |
| Splenic artery thrombosis | Paradoxical embolism |
| Splenic embolism | Paraneoplastic thrombosis |
| Stress cardiomyopathy | Paraparesis |
| Subclavian artery embolism | Paraplegia |
| Subclavian artery occlusion | Paresis |
| Subclavian artery thrombosis | Peripheral revascularisation |
| Thromboembolectomy | Pituitary infarction |
| Thrombotic microangiopathy | Placental infarction |
| Thrombotic thrombocytopenic purpura | Pneumatic compression therapy |
| Transient ischaemic attack | Portal shunt procedure |
| Truncus coeliacus thrombosis | Post procedural stroke |
| Vascular pseudoaneurysm thrombosis | Postpartum thrombosis |
| Vertebral artery occlusion | Prosthetic cardiac valve thrombosis |
| Vertebral artery thrombosis | Prosthetic vessel implantation |
| Visual acuity reduced transiently | Quadriparesis |
| Axillary vein thrombosis | Quadriplegia |
| Brachiocephalic vein occlusion | Renal infarct |
| Brachiocephalic vein thrombosis | Renal vascular thrombosis |
| Budd-Chiari syndrome | Retinal infarction |
| Catheterisation venous | Retinal vascular thrombosis |
| Cavernous sinus thrombosis | Shunt occlusion |
| Central venous catheterisation | Shunt thrombosis |
| Cerebral venous thrombosis | Spinal cord infarction |
| Compression garment application | Spinal stroke |
| Deep vein thrombosis | Splenic infarction |
| Deep vein thrombosis postoperative | Splenic thrombosis |
| Embolism venous | Stoma site thrombosis |
| Hepatic vein embolism | Stroke in evolution |
| Hepatic vein occlusion | Surgical vascular shunt |
| Hepatic vein thrombosis | Testicular infarction |
| Homans' sign positive | Thalamic infarction |
| Iliac vein occlusion | Thrombectomy |
| Inferior vena cava syndrome | Thromboangiitis obliterans |
| Inferior vena caval occlusion | Thrombolysis |
| Intracranial venous sinus thrombosis | Thrombosis |
| Jugular vein embolism | Thrombosis in device |
| Jugular vein occlusion | Thrombosis mesenteric vessel |
| Jugular vein thrombosis | Thrombosis prophylaxis |
| Mahler sign | Thrombotic cerebral infarction |
| May-Thurner syndrome | Thrombotic stroke |
| Mesenteric vein thrombosis | Thyroid infarction |
| Mesenteric venous occlusion | Tumour embolism |
| Obstetrical pulmonary embolism | Tumour thrombectomy |
| Obstructive shock | Tumour thrombosis |
| Ophthalmic vein thrombosis | Ultrasonic angiogram abnormal |
| Ovarian vein thrombosis | Ultrasound Doppler abnormal |
| Paget-Schroetter syndrome | Umbilical cord occlusion |
| Pelvic venous thrombosis | Umbilical cord thrombosis |
| Penile vein thrombosis | Vaccination site thrombosis |
| Phlebectomy | Vascular access site thrombosis |
| Portal vein cavernous transformation | Vascular device occlusion |
| Portal vein embolism | Vascular graft |
| Portal vein occlusion | Vascular graft occlusion |
| Portal vein thrombosis | Vascular graft thrombosis |
| Portosplenomesenteric venous thrombosis | Vascular operation |
| Post procedural pulmonary embolism | Vascular stent insertion |
| Post thrombotic syndrome | Vascular stent occlusion |
| Postoperative thrombosis | Vascular stent thrombosis |
| Postpartum venous thrombosis | Vasodilation procedure |
| Pulmonary embolism | Vessel puncture site occlusion |
| Pulmonary infarction | Vessel puncture site thrombosis |
| Pulmonary microemboli | Visual midline shift syndrome |
| Pulmonary thrombosis |  |

**Table S5.** SMQ “Hypertension” and PTs used according to MedDRA 22.0

| SMQ: Hypertension | |
| --- | --- |
| Accelerated hypertension | Renal sympathetic nerve ablation |
| Blood pressure ambulatory increased | Renovascular hypertension |
| Blood pressure diastolic increased | Retinopathy hypertensive |
| Blood pressure inadequately controlled | Secondary aldosteronism |
| Blood pressure increased | Secondary hypertension |
| Blood pressure management | Supine hypertension |
| Blood pressure orthostatic increased | Systolic hypertension |
| Blood pressure systolic increased | Withdrawal hypertension |
| Cardiometabolic syndrome | Aldosterone urine abnormal |
| Catecholamine crisis | Aldosterone urine increased |
| Diastolic hypertension | Angiotensin converting enzyme increased |
| Eclampsia | Angiotensin I increased |
| Endocrine hypertension | Angiotensin II increased |
| Essential hypertension | Angiotensin II receptor type 1 antibody positive |
| Gestational hypertension | Blood aldosterone abnormal |
| HELLP syndrome | Blood aldosterone increased |
| Hyperaldosteronism | Blood catecholamines abnormal |
| Hypertension | Blood catecholamines increased |
| Hypertension neonatal | Blood pressure abnormal |
| Hypertensive angiopathy | Blood pressure ambulatory abnormal |
| Hypertensive cardiomegaly | Blood pressure diastolic abnormal |
| Hypertensive cardiomyopathy | Blood pressure fluctuation |
| Hypertensive cerebrovascular disease | Blood pressure orthostatic abnormal |
| Hypertensive crisis | Blood pressure systolic abnormal |
| Hypertensive emergency | Catecholamines urine abnormal |
| Hypertensive encephalopathy | Catecholamines urine increased |
| Hypertensive end-organ damage | Diuretic therapy |
| Hypertensive heart disease | Ectopic aldosterone secretion |
| Hypertensive nephropathy | Ectopic renin secretion |
| Hypertensive urgency | Epinephrine abnormal |
| Labile hypertension | Epinephrine increased |
| Malignant hypertension | Labile blood pressure |
| Malignant hypertensive heart disease | Metanephrine urine abnormal |
| Malignant renal hypertension | Metanephrine urine increased |
| Maternal hypertension affecting foetus | Non-dipping |
| Mean arterial pressure increased | Norepinephrine abnormal |
| Neurogenic hypertension | Norepinephrine increased |
| Orthostatic hypertension | Normetanephrine urine increased |
| Page kidney | Pseudoaldosteronism |
| Postoperative hypertension | Renin abnormal |
| Pre-eclampsia | Renin increased |
| Prehypertension | Renin-angiotensin system inhibition |
| Procedural hypertension | Tyramine reaction |
| Renal hypertension |  |

**Table S6.** SMQ “Ischemic heart disease” and PTs used according to MedDRA 22.0

| SMQ: Ischemic heart disease | |
| --- | --- |
| Acute cardiac event | Coronary artery compression |
| Acute coronary syndrome | Coronary artery disease |
| Acute myocardial infarction | Coronary artery dissection |
| Angina unstable | Coronary artery insufficiency |
| Blood creatine phosphokinase MB abnormal | Coronary artery restenosis |
| Blood creatine phosphokinase MB increased | Coronary artery stenosis |
| Coronary artery embolism | Coronary artery surgery |
| Coronary artery occlusion | Coronary brachytherapy |
| Coronary artery reocclusion | Coronary bypass stenosis |
| Coronary artery thrombosis | Coronary endarterectomy |
| Coronary bypass thrombosis | Coronary no-reflow phenomenon |
| Coronary vascular graft occlusion | Coronary ostial stenosis |
| Kounis syndrome | Coronary revascularisation |
| Myocardial infarction | Coronary vascular graft stenosis |
| Myocardial necrosis | Diabetic coronary microangiopathy |
| Myocardial reperfusion injury | Dissecting coronary artery aneurysm |
| Myocardial stunning | ECG signs of myocardial ischaemia |
| Papillary muscle infarction | External counterpulsation |
| Periprocedural myocardial infarction | Haemorrhage coronary artery |
| Post procedural myocardial infarction | Ischaemic cardiomyopathy |
| Postinfarction angina | Ischaemic mitral regurgitation |
| Silent myocardial infarction | Microvascular coronary artery disease |
| Troponin I increased | Myocardial hypoxia |
| Troponin increased | Myocardial ischaemia |
| Troponin T increased | Percutaneous coronary intervention |
| Blood creatine phosphokinase abnormal | Prinzmetal angina |
| Blood creatine phosphokinase increased | Stress cardiomyopathy |
| Cardiac ventricular scarring | Subclavian coronary steal syndrome |
| ECG electrically inactive area | Subendocardial ischaemia |
| ECG signs of myocardial infarction | Wellens' syndrome |
| Electrocardiogram Q wave abnormal | Arteriogram coronary abnormal |
| Electrocardiogram ST segment abnormal | Cardiac stress test abnormal |
| Electrocardiogram ST segment elevation | Cardiopulmonary exercise test abnormal |
| Electrocardiogram ST-T segment elevation | Cardiovascular event prophylaxis |
| Electrocardiogram U wave inversion | Computerised tomogram coronary artery abnormal |
| Infarction | Elastic vessel recoil complication |
| Myocardial necrosis marker increased | Electrocardiogram PR segment depression |
| Scan myocardial perfusion abnormal | Electrocardiogram ST segment depression |
| Vascular graft occlusion | Electrocardiogram ST-T segment abnormal |
| Vascular stent occlusion | Electrocardiogram ST-T segment depression |
| Vascular stent thrombosis | Electrocardiogram T wave abnormal |
| Acute cardiac event | Electrocardiogram T wave inversion |
| Angina pectoris | Electrocardiogram U wave inversion |
| Angina unstable | Exercise electrocardiogram abnormal |
| Anginal equivalent | Exercise test abnormal |
| Arteriosclerosis coronary artery | Post angioplasty restenosis |
| Arteriospasm coronary | Restenosis |
| Coronary angioplasty | Stress echocardiogram abnormal |
| Coronary arterial stent insertion | Vascular stent stenosis |
| Coronary artery bypass | Wall motion score index abnormal |

**Table S7.** SMQ “Pulmonary hypertension” and PTs used according to MedDRA 22.0

| SMQ: Pulmonary hypertension | |
| --- | --- |
| Acute right ventricular failure | Banti's syndrome |
| Cardiac ventriculogram right abnormal | Bendopnoea |
| Central venous pressure increased | Brain natriuretic peptide increased |
| Chronic right ventricular failure | Cardiac cirrhosis |
| Cor pulmonale | Cardiac index decreased |
| Cor pulmonale acute | Cardiac murmur |
| Cor pulmonale chronic | Carotid pulse decreased |
| Portopulmonary hypertension | Combined pulmonary fibrosis and emphysema |
| Pulmonary arterial hypertension | Cyanosis central |
| Pulmonary arterial pressure abnormal | Diastolic dysfunction |
| Pulmonary arterial pressure increased | Dilatation ventricular |
| Pulmonary arterial wedge pressure increased | Dizziness exertional |
| Pulmonary artery dilatation | Dyspnoea |
| Pulmonary artery wall hypertrophy | Dyspnoea at rest |
| Pulmonary capillary haemangiomatosis | Dyspnoea exertional |
| Pulmonary endarterectomy | Dyspnoea paroxysmal nocturnal |
| Pulmonary hypertension | Exercise test abnormal |
| Pulmonary hypertensive crisis | Heart sounds abnormal |
| Pulmonary tumour thrombotic microangiopathy | Hepatojugular reflux |
| Pulmonary valve incompetence | Hypoxia |
| Pulmonary vascular resistance abnormality | Increased ventricular afterload |
| Pulmonary vein occlusion | Increased ventricular preload |
| Pulmonary vein stenosis | Intracardiac pressure increased |
| Pulmonary veno-occlusive disease | Irregular breathing |
| Right atrial dilatation | Jugular vein distension |
| Right atrial enlargement | Left ventricular end-diastolic pressure decreased |
| Right atrial hypertrophy | Non-cirrhotic portal hypertension |
| Right atrial pressure increased | Oedema due to cardiac disease |
| Right ventricular dilatation | Orthopnoea |
| Right ventricular dysfunction | Pulmonary arterial wedge pressure abnormal |
| Right ventricular enlargement | Pulmonary arteriopathy |
| Right ventricular failure | Pulmonary artery occlusion |
| Right ventricular heave | Pulmonary vascular disorder |
| Right ventricular hypertension | Respiratory fatigue |
| Right ventricular hypertrophy | Stroke volume decreased |
| Right ventricular systolic pressure increased | Tricuspid valve prolapse |
| Tricuspid valve incompetence | Tricuspid valve repair |
| Vascular resistance pulmonary increased | Tricuspid valve replacement |
| Abnormal precordial movement | Tricuspid valve sclerosis |
| Angiogram pulmonary abnormal | Tricuspid valve thickening |
| Atrial enlargement | Venous pressure jugular increased |
| Atrial pressure increased | Ventricular enlargement |

**Table S8.** SMQ “Torsade de pointes/QT prolongation” and PTs used according to MedDRA 22.0

| SMQ: Torsade de pointes/QT prolongation | |
| --- | --- |
| Electrocardiogram QT interval abnormal | Electrocardiogram U wave present |
| Electrocardiogram QT prolonged | Electrocardiogram U-wave abnormality |
| Long QT syndrome | Loss of consciousness |
| Long QT syndrome congenital | Multiple organ dysfunction syndrome |
| Torsade de pointes | Subacute kidney injury |
| Ventricular tachycardia | Sudden cardiac death |
| Cardiac arrest | Sudden death |
| Cardiac death | Syncope |
| Cardiac fibrillation | Ventricular arrhythmia |
| Cardio-respiratory arrest | Ventricular fibrillation |
| Electrocardiogram repolarisation abnormality | Ventricular flutter |
| Electrocardiogram U wave inversion | Ventricular tachyarrhythmia |

**Table S9.** Results of the disproportionality analysis in the MM-related indication subset*

| **Cardiovascular events** | **N** | **IC** | **IC_025_** | **IC_975_** | **ROR** | **ROR_025_** | **ROR_975_** |
| --- | --- | --- | --- | --- | --- | --- | --- |
| Total | 2774 | 0.52 | 0.46 | 0.58 | 1.53 | 1.46 | 1.59 |
| Cardiac arrhythmias | 540 | 0.58 | 0.43 | 0.72 | 1.52 | 1.39 | 1.65 |
| Cardiac failure | 631 | 0.95 | 0.82 | 1.08 | 2.00 | 1.84 | 2.16 |
| Cardiomyopathy | 1012 | 1.19 | 1.08 | 1.29 | 2.40 | 2.25 | 2.56 |
| Embolic and thrombotic events | 725 | -0.32 | -0.44 | -0.20 | 0.79 | 0.73 | 0.85 |
| Hypertension | 183 | 1.38 | 1.13 | 1.62 | 2.67 | 2.30 | 3.10 |
| Ischemic heart disease | 240 | 0.88 | 0.67 | 1.10 | 1.88 | 1.65 | 2.14 |
| Pulmonary hypertension | 479 | 1.77 | 1.62 | 1.92 | 3.60 | 3.28 | 3.95 |
| Torsade de pointes/QT prolongation | 273 | 0.74 | 0.54 | 0.94 | 1.70 | 1.51 | 1.92 |

*MM: multiple myeloma; N: number of records; IC_025_: the lower limit of a 95% CI for the IC; IC_975_: the upper limit of a 95% CI; ROR_025_: the lower limit of the 95%CI of ROR; ROR_975_: the upper limit of the 95%CI of ROR. IC_025_>0 and ROR_025_ >1 was deemed a signal.
